# Wdr18 Is Required for Kupffer's Vesicle Formation and Regulation of Body Asymmetry in Zebrafish

**DOI:** 10.1371/journal.pone.0023386

**Published:** 2011-08-18

**Authors:** Wei Gao, Linjie Xu, Rui Guan, Xinxing Liu, Yuxiang Han, Qian Wu, Yi Xiao, Fei Qi, Zuoyan Zhu, Shuo Lin, Bo Zhang

**Affiliations:** 1 Key Laboratory of Cell Proliferation and Differentiation of Ministry of Education, Center of Developmental Biology and Genetics, College of Life Sciences, Peking University, Beijing, People's Republic of China; 2 Department of Molecular, Cell & Developmental Biology, University of California Los Angeles, Los Angeles, California, United States of America; Instituto de Medicina Molecular, Portugal

## Abstract

Correct specification of the left-right (L-R) axis is important for organ morphogenesis. Conserved mechanisms involving cilia rotation inside node-like structures and asymmetric Nodal signaling in the lateral plate mesoderm (LPM), which are important symmetry-breaking events, have been intensively studied. In zebrafish, the clustering and migration of dorsal forerunner cells (DFCs) is critical for the formation of the Kuppfer's vesicle (KV). However, molecular events underlying DFC clustering and migration are less understood. The WD-repeat proteins function in a variety of biological processes, including cytoskeleton assembly, intracellular trafficking, mRNA splicing, transcriptional regulation and cell migration. However, little is known about the function of WD-repeat proteins in L-R asymmetry determination. Here, we report the identification and functional analyses of zebrafish *wdr18*, a novel gene that encodes a WD-repeat protein that is highly conserved among vertebrate species. *wdr18* was identified from a *Tol2* transposon-mediated enhancer trap screen. Follow-up analysis of *wdr18* mRNA expression showed that it was detected in DFCs or the KV progenitor cells and later in the KV at early somitogenesis stages. Morpholino knockdown of *wdr18* resulted in laterality defects in the visceral organs, which were preceded by the mis-expression of Nodal-related genes, including *spaw* and *pitx2*. Examination of morphants at earlier stages revealed that the KV had fewer and shorter cilia which are immotile and a smaller cavity. We further investigated the organization of DFCs in *wdr18* morphant embryos using *ntl* and *sox17* as specific markers and found that the clustering and migration of DFC was altered, leading to a disorganized KV. Finally, through a combination of *wdr18* and *itgb1b* morpholino injections, we provided evidence that *wdr18* and *itgb1b* genetically interact in the laterality determination process. Thus, we reveal a new and essential role for WD-repeat proteins in the determination and regulation of L-R asymmetry and propose a potential mechanism for *wdr18* in the regulation of DFC clustering and migration and KV formation.

## Introduction

Although the embryo appears bilaterally symmetrical during early development, molecular and cellular events occur asymmetrically to ultimately establish the left-right (L-R) axis in addition to the pre-existing anterior-posterior (A-P) and dorsal-ventral (D-V) axes. Initial symmetry-breaking events are critical for the L-R determination process, and involve genes encoding ion channels and ion pumps such as H^+^/K^+^-ATPase and H^+^-V-ATPase [1]–[3]. However, there are great differences in the mechanisms that govern the early establishment of asymmetry and more efforts are required to elucidate this process.

In mice, symmetry-breaking initiates in the node, which has equivalent structures in zebrafish (*Danio rerio*) (Kupffer's vesicle, KV), chicken (Hensen's node) and *Xenopus* (gastrocoel roof plate). Through the directional rotation of cilia inside the node or node equivalents, a leftward fluid flow is generated, which results in the asymmetric expression of Nodal-related gene *southpaw* (*spaw*), *lefty1*, *lefty2* and *pitx2* in zebrafish embryos [4]–[6]. In zebrafish, the KV is derived from dorsal forerunner cells (DFCs), which are a group of non-involuting cells located at the leading edge of the shield during gastrulation [4], [6]–[8]. DFCs first appear as dorsal surface epithelial cells that are in direct contact with the yolk syncytial layer and exhibit filopodia-like protrusions at the leading edge. At the onset of gastrulation, the DFCs start the process of clustering and migration toward the vegetal pole to form a compact cluster at the end of epiboly (bud stage). Afterwards, the DFCs give rise to KV structure through rapid epithelialisation and ciliogenesis during early somitogenesis. Meanwhile, the lumen volume and cilia length increase with the maturation of the KV [7].

Various genes and signaling pathways are necessary for the development of the KV and L-R asymmetry patterning. *ntl* is expressed in DFCs during gastrulation. Selectively knocking down *ntl* in DFCs results in a loss of the KV and laterality defects [9]. *polaris* and *pkd2* are important for ciliogenesis in the KV [10]. *fgf4* inhibition results in shorter cilia, indicating that Fgf signaling is also required for ciliogenesis in the KV [11]. Morpholino knockdown of *bmp4* also affects KV development [12]. A conserved role for Bmp signaling in controlling L-R patterning has also been discovered in other species [13]–[15].

Besides the specification of KV, migration and clustering of DFCs are also important processes for the proper formation of the KV. Defects in these steps result in a disorganized KV, which further leads to laterality abnormalities. However, the molecular mechanisms of these processes have not been examined until recently, and the genes and signaling pathways involved remain largely elusive. Combined inhibition of *wnt11* and *prickle1a* reduces DFC adhesion and impairs their organization and arrangement during KV lumen formation, which leads to the formation of a dysmorphic vesicle with small fragmented lumina and short cilia [16]. Knockdown of *integrin αV* or *β1b* causes defects in DFC migration, preventing the DFCs from organizing into a KV of normal shape and size [17]. However, how integrin signaling is processed and transmitted inside the cell during this process is still unknown.

Wdr18 is a member of the WD-repeat protein family which is found in all eukaryotes. The WD repeat consists of a 44 to 60 amino acid residue sequence that typically contains a GH dipeptide located 11 to 24 residues from its N-terminus and ends with a WD dipeptide at its C-terminus. Between the GH and WD dipeptides, there is a conserved core repeat sequence, which was first discovered in the β-subunit of hetero-trimeric G proteins [18]. Although different WD-repeat proteins belong to the same family, they have various functions. Zebrafish Oda/Wdr69 is recently shown to be required for axonemal dynein assembly and ciliary motility in ciliated organs including KV, knocking down of *wdr69* with morpholino leads to defects in asymmetric fluid flow inside KV and the establishment of organ laterality [19]. Currently, no functional data on *wdr18* is available and no WD-repeat proteins other than Wdr69 have been reported to be involved in L-R asymmetry.

The zebrafish has emerged as an excellent vertebrate model to study developmental processes, and it is especially advantageous for dissecting early embryogenesis, including the establishment of L-R asymmetry. Here, we identified and characterized *wdr18* as a novel gene involved in L-R determination through controlling the correct clustering and migration of DFCs. We also showed that *wdr18* and *itgb1b* interacted genetically, providing a possible mechanism for a role for *wdr18* in transducing integrin signaling in the context of laterality determination.

## Results

### Identification and expression pattern analysis of *wdr18*

We have performed a large-scale *Tol2* transposon-mediated enhancer-trap screen (unpublished data) in which mp235b (Figure S1) was identified as a transgenic fish line that trapped *wdr18*, a gene encoding a WD-repeat protein of 431 amino acids. Using the SMART program [20], [21] to analyze the Wdr18 protein sequence, we found it contains five WD repeats, which could be a potential interface for interacting with or binding to other proteins, and shares strong homology among vertebrate species (Figure S1).

**Figure 1:**
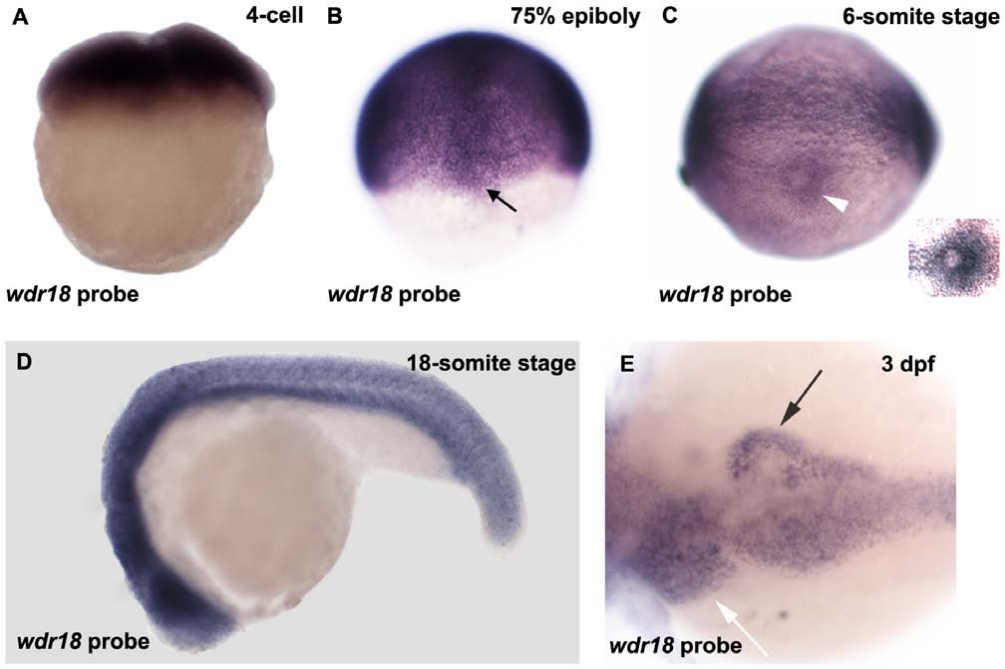
*wdr18* expression pattern during early embryonic development in zebrafish. (A) *wdr18* is maternally expressed. (B) Expression pattern of *wdr18* at the 75%-epiboly stage. Black arrow indicates the staining of dorsal forerunner cells (DFCs). (C) *wdr18* expression is detected around the Kupffer's vesicle (white arrowhead) at the 6-somite stage. Inset is an overexposed magnified image showing elevated *wdr18* expression around the KV. (D) *wdr18* is detected ubiquitously throughout the whole embryo at 18-somite stage. (E) At 3 dpf, *wdr18* is expressed in endodermal organs, including the exocrine pancreas (black arrow), liver (white arrow), and intestine.

Whole mount RNA *in situ* hybridization results showed that *wdr18* is maternally expressed from the 1–4 cell stage (Figure 1A) and in DFCs at 75%-epiboly stage (Figure 1B). By the 6-somite stage, its expression in the Kupffer's vesicle was detectable (Figure 1C) in addition to some basal expression levels in other cells. Later at the 18-somite stage, *wdr18* was detected ubiquitously throughout the embryo (Figure 1D). After 2 dpf (days post fertilization), *wdr18* was expressed in endodermal organs, including the pancreas and liver (Figure 1E), which is similar to the GFP expression pattern of mp235b (Figure S2).

### *wdr18* is required for the asymmetrical development of endodermal organs

**Figure 2:**
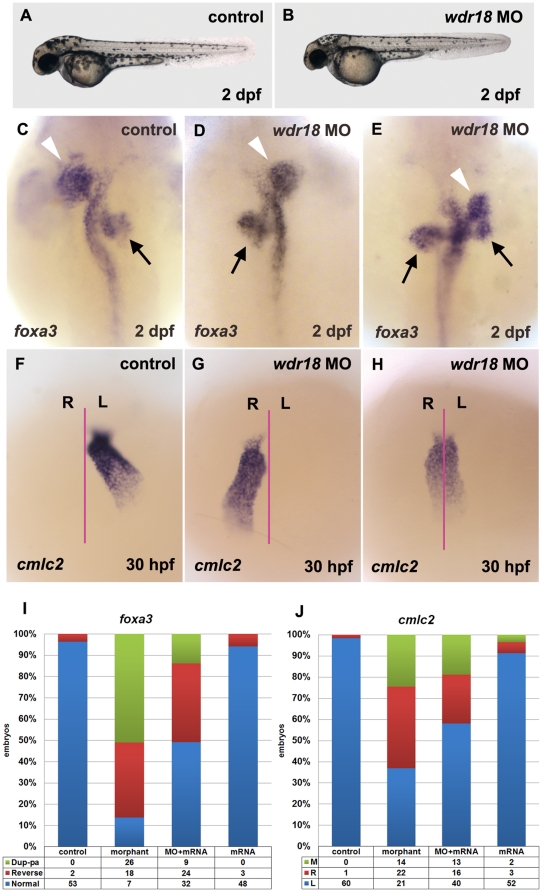
Knockdown of *wdr18* disturbed L-R asymmetry development of internal organs. (A, B) The overall appearance of a *wdr18* morphant embryo (B) is comparable to a wildtype control embryo (A). (C) Expression of *foxa3*, a marker for the endoderm, showed that the liver (white arrowheads) and pancreas (black arrows) were positioned to the left and the right of the midline in a control embryo at 2 dpf, respectively. In morphant embryos, the positions of the liver and pancreas were reversed (D), or the pancreas appeared duplicated (two black arrows) when the liver was randomized (E). *cmlc2*, a marker for the heart, was examined in embryos at 30 hpf. After the heart tube formation, leftward bending of the heart was observed in control embryos (F). By contrast, *wdr18* morphant embryos exhibited randomized movements: leftward, rightward (G), or no movement (H). The proportion of each phenotype is summarized in (I) and (J). Dup-pa in (I) indicates embryos with duplicated pancreas. M, R, and L in (J) stand for middle, right, and left, respectively. Injection of 100 pg *wdr18* mRNA partially rescued the endodermal organ phenotype. The statistical results are summarized in (I) and (J). (C–E) Dorsal views, anterior to the top. (F–H) Ventral views, anterior to the top.

Morpholinos are effective as antisense oligonucleotide analogs to knockdown gene expression in zebrafish [22]. To investigate the function of *wdr18*, we made use of two morpholinos against *wdr18*, MO1-I1E2 and MO2-E3I3, to interfere with *wdr18* mRNA splicing. Injection of either morpholino resulted in efficient exon losses and down-regulation of wild-type mRNA expression levels (E2 or E3 for MO1 and MO2, respectively; Figure S3). After injection of MO1 or MO2, the overall morphology of over 90% morphant embryos was relatively normal, except for a smaller head, bigger yolk sac with shorter yolk extension and shorter body length, which are the result of a general developmental delay commonly seen after morpholino injection (Figure 2A and 2B). We next examined internal organ development using the pan-endodermal marker *foxa3*. In control embryos at 2 dpf, the liver was located on the left side of the gut tube while the pancreas was on the right side (Figure 2C). In morphant embryos injected with either 4 ng MO1 or 16 ng MO2, the specification of endodermal organs was not affected; however, we observed a randomized distribution of these organs with the liver and pancreas appearing in opposite positions (Figure 2D) or in duplications (Figure 2E). The exocrine pancreas phenotype was further confirmed by *in situ* hybridization with *trypsin* (Figure S4E and S4F). Interestingly, the endocrine pancreas did not seem to be affected indicated by the staining result of *ins*, a marker for β cells (Figure S4C and S4D). A possible explanation for this phenotype is closely related to the mechanism of endocrine pancreas development. Endocrine pancreatic cells are known to be first specified around the midline as two bilaterally symmetric stripes of endodermal cells at 14 hpf (hours post fertilization), followed by migration toward the midline to form a compact cluster of endocrine cells [23]. Thus the initial differentiation of endocrine pancreas is symmetrical and likely independent of left-right determination process. These observations suggest that the L-R asymmetry is disturbed in *wdr18* morphants. In vertebrates, the heart is the most prominent indicator of proper L-R asymmetry; therefore, we examined the expression of the heart-specific marker *cmlc2* to determine whether heart asymmetry was affected. In control embryos at 30 hpf, the heart tube bent towards the left side (Figure 2F), which would subsequently morph into a characteristic “D loop” at later developmental stages. Knockdown of *wdr18* randomized the direction of tube bending with 36% of embryos displaying a leftward bend, 39% displaying a rightward bend (Figure 2G), and 25% remaining at the midline (Figure 2H). To confirm the specificity of this laterality phenotype, we injected 100 pg of *wdr18* full-length mRNA along with 4 ng MO1 or 16 ng MO2 and assayed for endodermal markers. We found that the laterality defect of visceral organs was partially rescued by *wdr18* over-expression (Figure 2I and 2J), while injection of 100 pg of *wdr18* full-length mRNA alone produced neither morphologically observable abnormal phenotype nor defects in left-right determination in the embryos (Figure 2I and 2J). The above results suggested that *wdr18* affects the L-R asymmetry of internal organs, but not their specification.

### *wdr18* regulates expression of genes that determine L-R asymmetry

**Figure 3:**
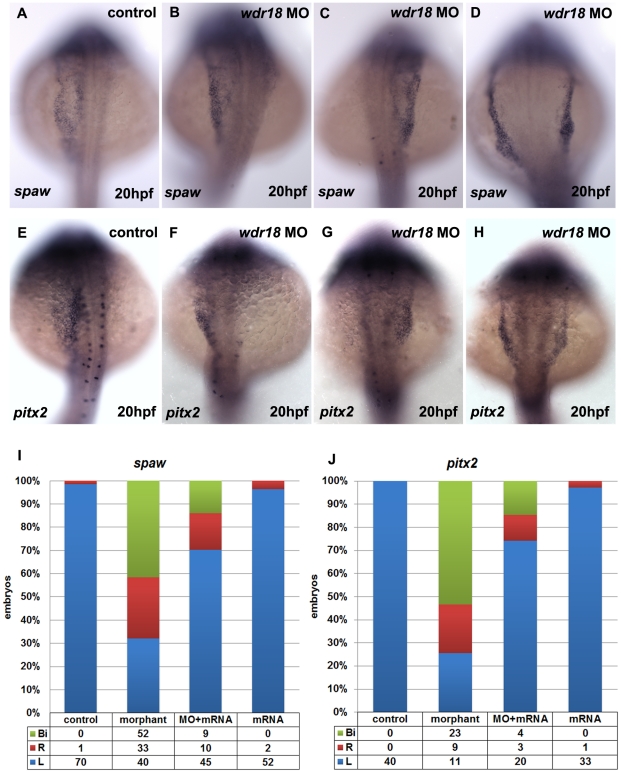
*wdr18* morpholino caused randomized expression of *spaw* and *pitx2*. In nearly all control embryos, *spaw* and *pitx2* showed left-sided expression (A, E), whereas *wdr18* morphants showed left-sided (B, F), right-sided (C, G), or bilateral (D, H) *spaw* or *pitx2* expression in the LPM. (I, J) The percentage of control, morphant, mRNA-rescued and mRNA over-expressed embryos with left-sided (L), right-sided (R), or bilateral (Bi) *spaw* and *pitx2* expression. (A–H) Dorsal views, anterior to the top.

To address the mechanism of the above laterality phenotype, we checked markers that are specifically expressed on the left side of the embryo [24]–[26]. At the 19- to 21-somite stage, changes in the expression of the Nodal-related gene *spaw* were found in *wdr18* morphants (Figure 3A–D). In un-injected control embryos, *spaw* was expressed in the left LPM (lateral plate mesoderm) in about 99% of embryos (Figure 3A), whereas in *wdr18* morphants, *spaw* expression was randomized, with 32% of the embryos exhibiting normal left-sided expression (Figure 3B), 26% showing right-sided expression (Figure 3C), and 42% showing bilateral expression (Figure 3D).

*pitx2* is a downstream effector of the Nodal signaling cascade. Examination of *pitx2* expression showed that it was altered in *wdr18* morphants (Figure 3E–H). Nearly all of the un-injected control embryos showed normal left-sided expression of *pitx2* in the LPM (Figure 3E), while in *wdr18* morphants, the expression pattern was randomized, with 25% of embryos showing left-sided expression, 21% showing right-sided expression, and 54% showing expression on both sides. By injecting *wdr18* full-length mRNA, the misexpression of *spaw* or *pitx2* in the LPM could partially be rescued (Figure 3I and 3J), confirming the specificity of *wdr18* in regulating the expression of L-R determination genes like *spaw* and *pitx2* in the LPM. Then we reached a conclusion that *wdr18* knockdown disrupts the L-R specification process in the early-staged embryos.

### *wdr18* is required for the formation and function of the Kupffer's vesicle

**Figure 4:**
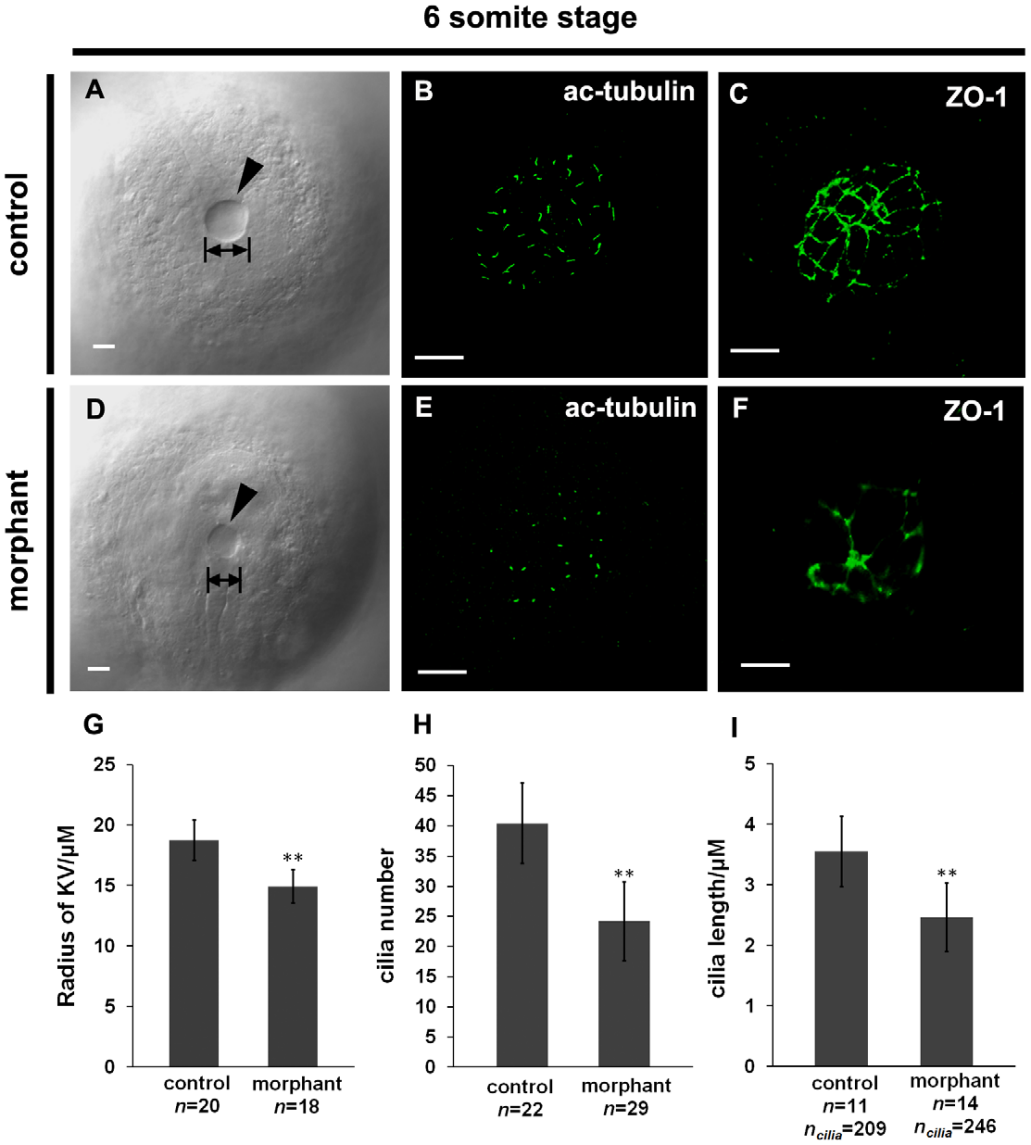
KV formation is disturbed in *wdr18* morphant embryos. (A, D) DIC images of KV in control (A) and *wdr18* morphant (D) embryos. Note the KV size in control was larger than that in the morphants. (B, E) Confocal images of cilia inside KV in control (B) and morphant (E) embryos detected by a fluorescent anti-acetylated tubulin antibody. Shown is a 3D projection of multiple focal slices spanning KV at an interval of 0.4 µm. (C, F) anti-ZO-1 staining of the KV in 6- to 8-somite stage embryos. The control embryo (C) developed a KV with a large fluid-filled lumen, showing a uniform ZO-1-positive tight junction lattice within the lining of the KV, while the *wdr18* morphant embryo (F) showed a dysmorphic lattice of ZO-1 labeling within the KV. (G) Measurement of the radius of individual KV in control and morphant embryos at 6- to 8-somite stage and statistical analyses. (H) Graphic representation of quantification results of cilia number per KV in both control and morphant embryos at 6- to 8-somite stage. (I) Quantification result of cilia length in control and *wdr18* morphant embryos at 6- to 8-somite stage. The double asterisks in G, H and I indicate that the difference is extremely significant between experimental and control samples (*P*<0.0001, Student's *t*-test). Scale bars: 20 µm in A and D; 15 µm in B, C, E and F.

The Kupffer's vesicle is a ciliated organ formed during zebrafish embryogenesis, and is important for the establishment of L-R asymmetry. Through the oriented rotation of cilia inside the KV, the asymmetric expression of genes, including *spaw*, *pitx2*, *lefty1* and *lefty2*, is established. *In situ* hybridization analysis revealed that *wdr18* is expressed in DFCs and later in the KV (Figure 1B and 1C), which implies that *wdr18* is required for KV function. Under a light microscope, the KV was much smaller in *wdr18* morphant embryos than in control embryos (mean radius in control embryos, 18.45±1.38 µm, s.d.m, *n* = 20; mean radius in morphants, 14.94±1.64 µm, s.d.m, *n* = 15, *p*<0.0001) (Figure 4A, 4D and 4G). To better evaluate the KV phenotype, we used anti-acetylated tubulin to visualize the cilia inside the KV. Compared with control embryos, the number of cilia within the KV was significantly reduced in the *wdr18* morphants. The mean number of cilia per KV was 40.5±6.7 in control embryos (*n* = 22) while in *wdr18* morphants the number was reduced to 24.2±6.6 (*n* = 29). (Figure 4B, 4E and 4H). We found that the cilia length was also significantly reduced in *wdr18* morphants (2.46±0.56 µm, s.d.m; *n* = 14 embryos, *n*_cilia_ = 246; *p*<0.0001) compared with that in control embryos (3.55±0.58 µm, s.d.m; *n* = 11 embryos, *n*_cilia_ = 209) (Figure 4I). We then used ZO-1 antibody, a marker for tight junctions between cells, to visualize the structure of the KV. We observed that the cells composing KV in morphant embryos did not show tight connection to form a uniform lattice as in control embryos (Figure 4C and 4F). These results indicate that KV formation is disturbed in *wdr18* morphants.

**Figure 5:**
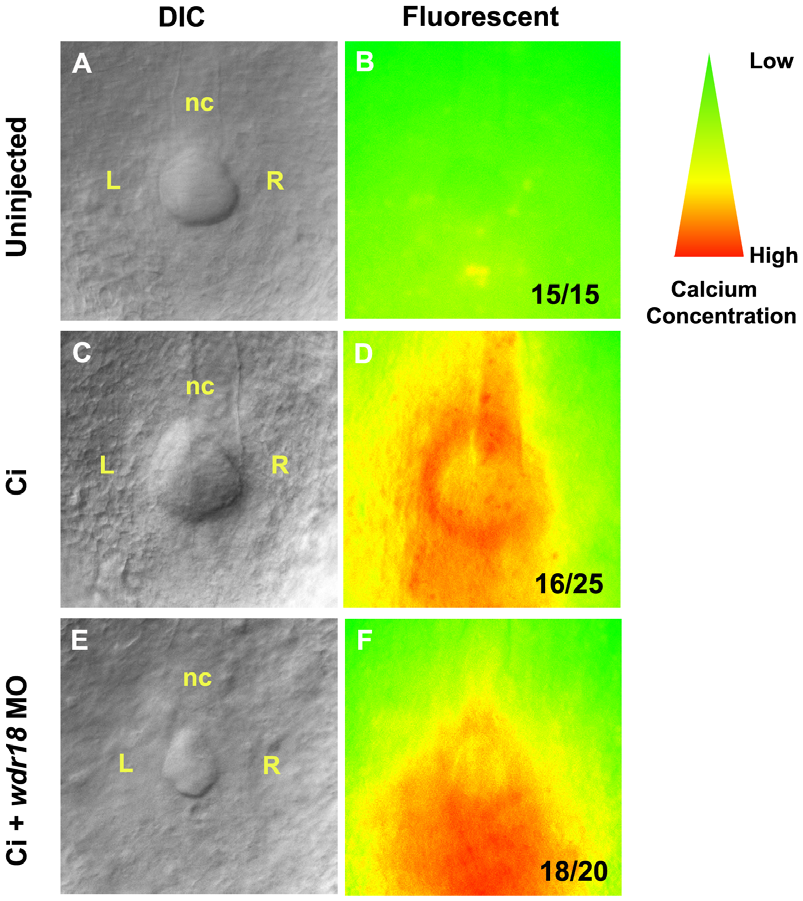
Correct Ca^2+^ distribution in the KV during early somite stages requires *wdr18*. (A–F) Embryos were injected at the 1-cell stage with Ca^2+^ indicator (Ci) or co-injected with *wdr18* MO1. Ca^2+^ patterns at the 5- to 8-somite stage were imaged by fluorescence microscopy and the images were converted to an intensity scale (colors from red to green indicate intensity from high to low). (A, B) The autofluorescent image of an un-injected control embryo (B) with its corresponding DIC image (A). (C, D) A control embryo injected with Ca^2+^ indicator only, showing fluorescence in the KV with elevated signals on the left side. (E, F) An embryo co-injected with Ca^2+^ indicator and *wdr18* MO1. The KV is smaller and the fluorescence signal is evenly distributed throughout the KV. nc: notochord. The ratio shown in the lower right corner of B, D and F indicate the number of defective embryos versus total embryos.

Through directional rotation of cilia inside the KV, ions like calcium are asymmetrically distributed with higher concentrations on the left side. To further confirm that the function of the disorganized KV was affected, we checked the distribution of calcium by injection of a Ca^2+^ indicator (Ci) as previously reported [27], [28]. The intensity of its fluorescent signal is proportional to the calcium concentration in the environment. In control embryos, we found significantly elevated calcium concentrations on the left side of the KV in 16 out of 25 embryos examined (Figure 5C and 5D), whereas in *wdr18* morphant embryos, the calcium signal was weaker and evenly distributed throughout the KV in 18 out of 20 embryos (Figure 5E and 5F).

The asymmetric distribution of calcium is dependent on directional fluid flow generated by the cilia rotation inside KV. So we next examined whether the fluid flow was affected in the KV of morphant embryos. By injection of fluorescent microspheres (diameter = 0.5 µm) into the KV of both control and morphant embryos at 3- to 6-somite stage, we consistently observed that the counter-clockwise rotation of the beads as found in control embryos was abolished in morphant embryos (movies S1 and S2). This result further support our conclusion that the KV function was affected when *wdr18* was down regulated.

### *wdr18* is involved in the migration and organization of DFCs

**Figure 6:**
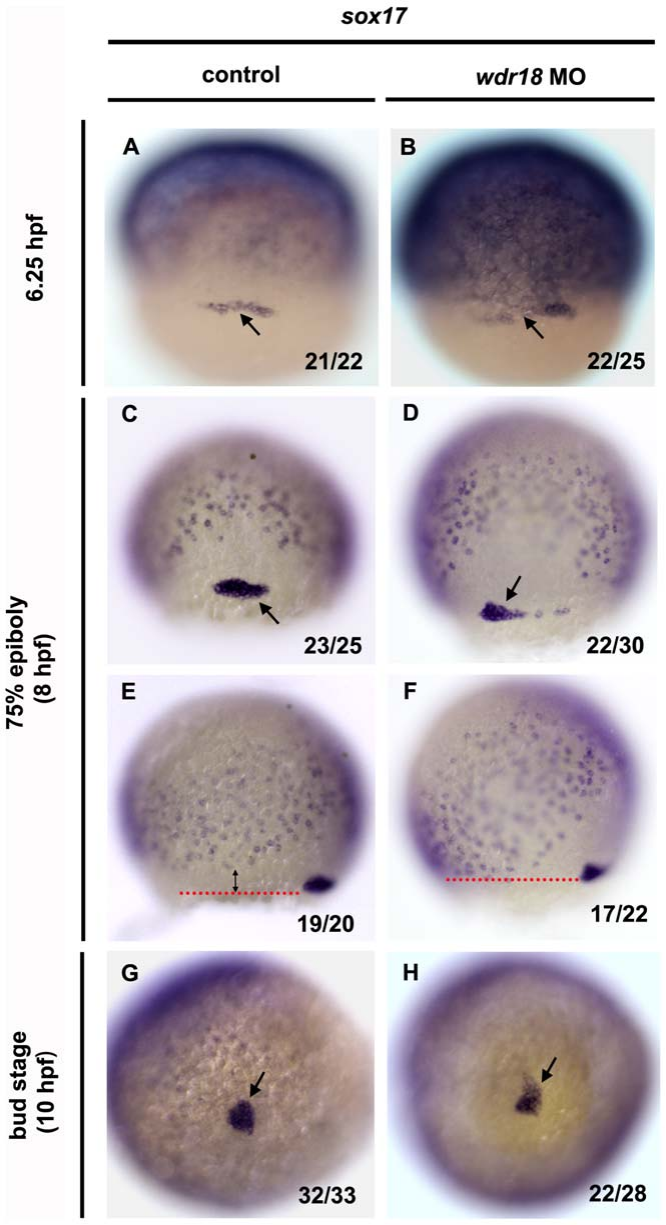
DFC migration is affected in *wdr18* morphant embryos. (A–H) *In situ* hybridization with *sox17* showing the migration and clustering process of DFCs in control and *wdr18* morphant embryos at 6.25 hpf, 75%-epiboly stage and bud stage. *sox17* is a marker of endodermal cells and DFCs. The pepper-like staining represents endodermal cells and the grouped cells (black arrow) indicate DFCs. (A, C, G) The DFCs in control embryos first appeared as a horizontal line of cells (A), and then migrated toward the vegetal pole while forming a compact oval-shaped cluster (C) and eventually a round condensed cell mass at the end of epiboly, which will gradually differentiate into the Kupffer's vesicle. (B, D, H) In *wdr18* morphant embryos, the shape of DFCs looked nearly the same as control embryos at 6.25 hpf (B), however, the DFCs failed to form a single compact cell cluster at 75%-epiboly stage (D). Although the DFCs in morphant embryos could finish clustering process at the end of epiboly, the cell mass was frequently appeared misshaped (H). (E, F) The location of the DFCs relative to the endodermal cell layer. (E) In a control embryo, the DFC migratory front (red dotted line) is further ahead of the endodermal cell layer as a result of DFC migration towards the vegetal pole. (F) In the morphant embryo, the DFC migratory front is almost at the same level as the endodermal layer, indicating that DFC migration is slower and uncoupled with overall gastrulation movements. The ratio shown in the lower right corner of each image indicate the number of defective embryos versus total embryos. (A–D) Dorsal views, animal pole to the top. (E, F) Side views, dorsal to the right, animal pole to the top.

The precursors of ciliated KV cells are dorsal forerunner cells, a group of 20–30 non-involuting cells in the zebrafish gastrula. We next investigated whether DFCs were affected in *wdr18* morphants by examining the expression of *sox17*, a marker for endodermal progenitors and DFCs, to visualize the behaviors of individual DFC cells. Knockdown of *sox17* leads to a smaller KV with fewer cilia [29]. In control embryos, the DFCs first appeared as a horizontal line of scattered cells, which later went through clustering and migration process toward the vegetal pole to form a compact oval-shaped cluster of cells and eventually a condensed cell mass at the tail bud region at the end of epiboly (Figure 6A, 6C and 6G). In *wdr18* morphant embryos, the clustering and migration of the DFCs was disturbed, with cells rarely form clusters during epiboly (Figure 6B) and turn into a malformed cell mass at the bud stage (Figure 6H). Our observation that DFCs in *wdr18* morphant embryos could finally migrate to the vegetal pole indicated that the migration of DFC was not totally disrupted. However, we found that during epiboly, DFCs in control embryos remained ahead of cell layers undergoing epiboly and involution (Figure 6E) while DFCs in morphants exhibited slower migration towards the vegetal pole and therefore uncoupled with cells in other layers (Figure 6F). We also used *ntl* to detect the DFCs and found that its staining in the DFC region was also altered in morphants (Figure S5A and S5B). Double *in situ* hybridization with *ntl* and *sox17* confirmed the co-staining as well as the altered pattern of the two genes in DFCs, indicating that knocking down of *wdr18* lead to mis-localization but not abnormal specification of the DFCs, resulting in their clustering and migration defect (Figure S5C and S5D). These results suggest that *wdr18* is involved in the regulation of the KV formation through directing the correct migration and organization of DFCs.

### *wdr18* and *integrin β1b* act synergistically in left-right asymmetry determination

**Figure 7:**
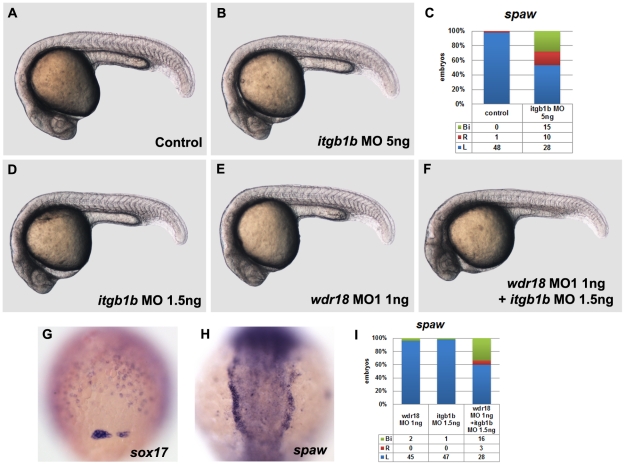
*wdr18* and *integrin β1b* act genetically in the regulation of laterality development. (A) A control embryo under a light microscope. (B) The overall morphology of embryos injected with 5 ng of *itgb1b* morpholino is indistinguishable from controls. (C) The percentage of control and *itgb1b* morphant embryos with left-sided (L), right-sided (R), or bilateral (Bi) *spaw* expression. (D, E, F) The morphology of embryos injected with 1.5 ng *itgb1b* morpholino (D) or 1 ng *wdr18* MO1 (E), and embryos injected with a combination of 1 ng *wdr18* MO1 and 1.5 ng *itgb1b* morpholino (F) look relatively normal, except for a minor difference in body curvature (F). (G) *sox17* staining in embryos injected with a combination of 1 ng *wdr18* MO1 and 1.5 ng *itgb1b* morpholino indicates that DFC migration is affected. Dorsal view, animal pole to the top. (H) An example of bilateral *spaw* expression in the LPM of embryos injected with a combination of 1 ng *wdr18* MO1 and 1.5 ng *itgb1b* morpholino. Dorsal view, anterior to the top. (I) The percentage of control embryos injected with 1.5 ng *itgb1b* morpholino, 1ng *wdr18* MO1, or a combination of 1 ng *wdr18* MO1 and 1.5 ng *itgb1b* morpholino showing left-sided (L), right-sided (R), or bilateral (Bi) *spaw* expression.

The integrin signaling pathway mediates cell-cell and cell-extracellular matrix interactions, making it an important pathway in animal embryonic development. There are eighteen α and eight β integrin subunits in mammals, which can assemble into 24 different heterodimers. Among the subunits, *integrin αV* and *β1b* have recently been reported to be involved in the L-R determination process by controlling the migration of DFCs [17]. When 5 ng *integrin β1b* morpholino was introduced into the zebrafish embryos, normal left-sided *spaw* expression was disrupted (Figure 7A–C). Furthermore, we observed a DFC migration defect similar to that of *wdr18* morphants (Figure 6D), which led us to suspect that *wdr18* and *integrin β1b* might have functional interactions. To explore this possibility, we co-injected *wdr18* MO1 and *integrin β1b* morpholino into the same embryos. When *wdr18* MO1 and *integrin β1b* morpholino were delivered separately at lower doses, 1 ng and 1.5 ng respectively, no laterality defects were observed (Figure 7D, 7E and 7I). However, when both morpholinos were injected together into the same embryos, DFC migration was affected (Figure 7G) and the L-R asymmetry was disturbed (Figure 7H and 7I), while the overall morphology of the morphants remained relatively normal (compare Figure 7D with 7F). Based on these results, we conclude that *wdr18* and *integrin β1b* act genetically together to control the formation of the KV and determine L-R asymmetry.

## Discussion

### Wdr18 is a new member of WD-repeat protein family identified to control left-right asymmetry determination

In this study, we identified *wdr18* as a novel WD-repeat family gene that plays an important role in the establishment of L-R asymmetry. By knocking down *wdr18*, we showed that all visceral organs were normally specified in *wdr18* morphants, but they emerged on opposite sides of the gut tube or in a duplicated form. To understand the molecular events underlying this phenotype, we further showed that early L-R determination events were affected; specifically, the expression of Nodal-related gene *spaw* and *pitx2* were randomized in the LPM. The KV was also malformed, exhibiting a significant reduction in its size as well as the length and number of its cilia. Next, we established that *wdr18* was required for the correct clustering and migration of DFCs and the normal specification of the L-R axis. Through careful examination on the images of KV obtained through confocal 3D reconstruction as well as conventional fluorescence microscope revealed by immunohistochemical staining with ZO-1, we found that the size of the cells in the KV showed no obvious difference in both control and morphant embryos (Figure S6), except that the connections between cells in the morphants was not as tight and regular as those in control embryos (Figure 4C and 4F). This observation suggests that the KV became smaller in *wdr18* morphants is probably due to there are fewer rather than smaller cells. After careful examination on the cilia distribution inside KV under confocal microscope, we could see the distribution of cilia in *wdr18* morphants is even and similar to the density in control embryos, though both the cilia number and length were reduced. So it is likely that the reduction of cilia number in KV is due to a reduction of the cell number in KV after *wdr18* down regulation. However, there seems no obvious reduction in the intensity of the staining of *sox17* mRNA in *wdr18* morphants at the bud stage (Figure 6G and 6H), which implies that the number of DFC cells that give rise to KV was probably not affected at that stage after knocking down *wdr18*. This raises the possibility that the migration and clustering defects of DFCs lead to fewer cells to develop normally to form a functional KV. From Figures 6G and 6H, we could see the shape of *sox17* positive DFCs is irregular and this defect could potentially lead to the malformation of KV structure in early somitogenesis stages, which is known to be a well orchestrated process [7]. In other words, the reduction of KV volume, cilia number, cilia length and the defects in cilia motion could be the direct result of the DFC migration and clustering defect in *wdr18* morphants. Nevertheless, we can not rule out the possibility that *wdr18* was directly involved in the ciliogenesis process, given that Oda/Wdr69, another WD repeat family protein, was reported to be required for axonemal dynein assembly and ciliary motility in KV [19].

### A comparison between the GFP fluorescent signal in mp235b transgenic fish and *wdr18* mRNA expression pattern

From 30 hpf and thereafter, GFP was expressed in the endodermal organs of mp235b transgenic fish. Besides the pancreatic region, GFP was expressed transiently in the liver and foregut at 2 dpf, while it was restricted to the exocrine pancreas from 3 dpf. *In situ* hybridization analysis of *wdr18* mRNA expression pattern showed that *wdr18* was expressed constantly in the liver and foregut (Figure S2D–F, black arrows) from 2 dpf. The expression pattern difference between GFP fluorescence and *wdr18* RNA *in situ* hybridization indicated that the enhancer trapped by the GFP insertion (100 kb upstream) may only partially reflect the real transcriptional regulatory region of the *wdr18* gene. Interestingly, although *wdr18* was ubiquitously expressed during gastrulation and segmentation, its expression level was elevated around the KV in 6- to 8-somite stage embryos, and showed clear staining of DFCs in the 75%-epiboly stage embryos, indicating that the L-R asymmetry defect was the primary result of *wdr18* knocking down by morpholino injections. In this case, the *wdr18* mRNA expression pattern could be categorized into two phases: an early expression phase that includes DFCs and the KV, and a later phase that restricts its expression to endodermal organs.

### A possible mechanism for *wdr18* in the regulation of DFC clustering and migration

*Immediate early response 2* (*Ier2*) and *fgf intra-cellular binding protein 1* (*fibp1*) are downstream effectors of *fgf8*, with ubiquitous expression patterns at early embryonic stages. Knockdown of these two genes causes laterality defects by disturbing KV formation following convergence and extension defects [30]. Knockdown of *wdr18* may also cause mild convergence and extension defects, which would further lead to the abnormal migration and organization of DFCs. It is possible that *wdr18* is involved in pathways that control gastrulation movements, which then globally control the behavior of DFCs. *integrin αV* and *β1b* are required for DFC migration. Knockdown either of them results in L-R asymmetry defects [17]. In eukaryotic cells, the WD-repeat protein Rack1 binds to protein kinase C and integrin β1 and β2, linking PKC directly to integrins and participating in the regulation of integrin signaling pathways [31], [32]. In a previous yeast two-hybrid screen, WD protein associating with integrin cytoplasmic tails 1 (WAIT-1), a human WD-repeat protein, was shown to interact with the cytoplasmic tail of integrin β7, potentially acting as a regulator of integrin functions [33]. These reports suggest that some WD-repeat proteins bind to integrins and mediate downstream pathways. Based on these data and our results from co-injecting both *wdr18* and *integrin β1b* morpholinos, we hypothesize that *wdr18* functionally interacts with the integrin pathway to control DFC migration; however, more evidence is necessary to determine whether Wdr18 directly binds to integrin β1b.

## Materials and Methods

### Zebrafish maintenance

Zebrafish were raised and kept under standard laboratory conditions at 28.5°C. Embryos were staged according to Kimmel *et al* [34]. mp235b fish were generated from a previous *Tol2* transposon-based enhancer trap screen (unpublished data). The wild-type line used was AB.

All animal experiments were approved by Institutional Animal Care and Use Committee (IACUC) of Peking University. The reference from IACUC of Peking University is LSC-ZhangB-1.

### Whole-mount *in situ* hybridization

The whole mount *in situ* hybridization with digoxigenin or fluorescin labeled ribo-probe was performed essentially as previously described [35]. NBT/BCIP (50 mg/mL; Promega) or Fast Red (Roche) was used as alkaline phosphatase substrates. The following probes, which were synthesized previously and kept in our lab, were used: *wdr18*, *pdx1*, *insulin*, *trypsin*, *foxa3*, *cmlc2*, *spaw*, *pitx2*, *sox17*, *ntl*.

### RNA synthesis and injection

Total RNA was isolated from 1 dpf embryos using the Trizol (Invitrogen) method. *Wdr18* full-length coding sequence was amplified by RT-PCR (94°C for 30 sec, 55°C for 30 sec, and 72°C for 90 sec, for 30 cycles) with the following primers: 5′- GCGCTCGAGAACATGTCGGCGCCCGT- 3′ and 5′- GCGACTAGTGAGAACAATCCTGCCCTCAGG- 3′, then subcloned into pXT7 vector. The corresponding mRNAs were synthesized using the T7 mMessage mMachine kit (Ambion). mRNA injection was performed at the one-cell stage as described [36]. Sterile water was used for the control experiments. One hundred picograms of *wdr18* mRNA was used for all experiments.

### Design and injection of antisense morpholino oligonucleotides

Antisense morpholino oligos (MOs) designed against *wdr18* targeting I1E2 (MO1) or E3I3 (MO2) and *integrin β1b* targeting I9E10 [17] were obtained from Gene Tools and injected into one- to two-cell stage embryos [37]. All morpholinos were prepared and resuspended in 1× Danieau's buffer at 4 ng/nL for injection.

Sequence information is as follows:

*wdr18*-I1E2-MO1: TGTAGCTGGTCCTGAAAAGAAGTTT

*wdr18*-E3I3-MO2: ATGAAGGGCTTATAACAGACCTGGA

To analyze the effect of *wdr18*-I1E2-MO1 and *wdr18*-E3I3-MO2 on mRNA splicing, the following primers were used.

Primer pair e1 (5′-GCTGTTTAACTGTGCGGTTTATGA -3′) and e3 (5′-AAGCCAGATTGTCTTTCCCTCC -3′) amplify a product of normal splicing or exon2 skipping; primer pair e2 (5′-GAGATCCAAAGGAAGGACCAGC -3′), and e4 (5′-CAGAACAGAAAATACTCGCAGGG-3′) amplify fragments of normal splicing or exon3 skipping. The optimal concentration to reach a sufficient knockdown effect was 4 ng per embryo for MO1 and 16 ng per embryo for MO2.

*integrin β1b* MO: 5′-GCCAGTTTGAGTGAATAACTCACCT-3′.

### Whole-mount immunohistochemistry

Immunohistochemical (IHC) staining was performed according to a standard protocol [10]. The antibodies used were as follows: mouse anti-ZO-1 (Invitrogen, Cat No. 339100) at 1∶400, and mouse monoclonal anti-acetylated tubulin antibody (Sigma, Cat No. T7451) at 1∶400. Alexa Fluor 488-conjugated anti-mouse IgG was used as secondary antibody (Invitrogen). Whole embryos were then observed under a confocal microscope.

### Detection of intracellular Ca^2+^ in zebrafish embryos

Ca^2+^ green-dextran 10,000 MW (Molecular Probes/Invitrogen) was used at 0.05% (w/v) for microinjection into zebrafish embryos at the 1-cell stage with an injection volume of about 0.5 nL. Fluorescent images at 400 ms exposure of the KV at the 6-somite stage were captured using the Axioimager Z1 fluorescence microscope (Zeiss) and the intensity of images was measured using ImagePro+ software (Media Cybernetics).

### Imaging and processing

Images of *in situ* hybridization were captured using a digital camera (Axiocam) attached to a compound Zeiss Axioimager A1 microscope and processed by Photoshop (Adobe) software. Confocal *z*-series images were captured using a Zeiss LSM 710 Laser Scanning Confocal Microscope or a Leica SP5 Confocal Microscope, and then visualized and analyzed with ImageJ (NIH) software. The length of all cilia in each KV was measured using ImageJ Freehand Line Tool from 3D projections of *z*-stacks acquired with a space of 0.4 µm. The total number of cilia within KV was determined from *z*- projections of confocal *z*-stacks spaced at 1.78 µm.

### Fluorescent microspheres injection

Embryos were removed from their chorions and placed in the injection mold previously covered with 2.5% methylcellulose and fish water. The FluoSpheres® fluorescent microspheres (Molecular Probes, 0.5 µm) were injected into KV at the 6-somite stage under oil pressure. Embryos were imaged under a Zeiss Axioimager Z1 fluorescence microscope with a 20×Plan Neofluar objective. Movies were taken with a digital camera (Axiocam) attached to the microscope and processed with VirtualDub software.

## Supporting Information

Figure S1 **Sequence alignment and structure of Wdr18 protein.** (A) Alignment of human, mouse and zebrafish Wdr18 peptide sequences. The amino acid residues that are identical in all three species are shaded in dark blue and those conserved in just two of them are shaded in light blue. Red horizontal lines below the sequences indicate the location of WD-repeats. (B) The schematic structure of zebrafish Wdr18 protein with black boxes indicating the location of the five WD repeats. The alternating dark purple and light purple boxes below indicate coding regions of exons 1–10 and the relative positions to the five WD-repeats.
(TIF)

Figure S2 **GFP fluorescent signal in mp235b embryos and** ***wdr18*** **expression in endodermal organs.** (A–C) GFP expression pattern in mp235b transgenic line. (D–F) Whole-mount *in situ* hybridization showing *wdr18* gene expression in endodermal region during the first 3 days of development. Arrowheads indicate pancreas and arrows indicate liver region. All the embryos were shown as dorsal views with anterior to the left.
(TIF)

Figure S3 **Injection of MO1 and MO2 against** ***wdr18*** **result in efficient exon loss and mRNA down-regulation.** (A) Illustration of the structure of *wdr18* from 1^st^ to 4^th^ exons and the target sites for MO1 and MO2. (B) Introducing MO1 into embryos result in exon 2 skipping, or an 111 nt deletion of *wdr18* mRNA, which causes the loss of the first WD repeat of Wdr18 protein. (C) Injection of MO2 leads to exon 3 skipping, or a 134 nt deletion, causing a frameshift for the sequence after exon 3. The total RNA were extracted from embryos at 1 dpf.
(TIF)

Figure S4 **The effect of** ***wdr18*** **knockdown on endodermal organs.** (A, B) *pdx1* was used as a marker for the pancreatic bud. In morphant embryos, the pancreas appeared on both sides of the body (B), while in control embryos it resided on the right side. (C, D) *insulin* (*ins*) was used as a differentiation marker for pancreatic β cells. Note that the development of β cells was not affected after *wdr18* morpholino injection. (E, F) *In situ* hybridization of *trypsin* (*try*) revealing the exocrine pancreas at 4 dpf. In morphant embryos, the exocrine pancreas differentiated normally, but appeared in duplicated form. Dorsal views, anterior to the top.
(TIF)

Figure S5 **Knockdown of** ***wdr18*** **led to defects in DFC migration without affecting DFC specification.** (A) *In situ* hybridization result of *ntl*, showing the notochord precursor cells at the midline, marginal cells, and DFCs (black arrowhead) in a control embryo at 60%-epiboly stage. (B) The midline structure and marginal cells were not affected, while the DFC region, marked by a white arrowhead, seemed reduced or mis-localized in a *wdr18* morphant. (C, D) Double *in situ* hybridization result showed clear staining of both *ntl* (red) and *sox17* (blue) positive cells in *wdr18* morphant embryos, indicating DFCs were still present after knockdown of *wdr18*, although they were not properly organized as a single cluster. Dorsal views, animal pole to the top.
(TIF)

Figure S6 **Knockdown of** ***wdr18*** **did not change the size of cells composing Kupffer's vesicle.** 3D reconstruction confocal images of the KV in control and *wdr18* morphant embryos revealed by immuno-staining with ZO-1 antibody at 6-somite stage. For the convenience of outlining the cell shape, the 3D images were rotated to the proper position until the boundaries of the marked cells was clearly seen. (A) In a control embryo, the cell connection was obvious and we outlined four cells with different colors and numbered them from 1 to 4. (B) In a *wdr18* morphant, we similarly outlined and numbered four cells for comparison. The size of the marked cells showed no obvious difference comparing with that in control embryos. Scale bars: 15 µm.
(TIF)

Movie S1 **A wild type control embryo with its KV injected with the 0.5 µm fluorescent microspheres.** Note the counter-clockwise rotation of the beads.
(MOV)

Movie S2 **A** ***wdr18*** **morphant embryo with its KV injected with the 0.5 µm fluorescent microspheres.** Note the beads only move slightly and irregularly.
(MOV)
