# Supplementary figures and images for: Wdr18 Is Required for Kupffer's Vesicle Formation and Regulation of Body Asymmetry in Zebrafish
Source: PLoS One. 2011 Aug 18;6(8):e23386. doi: 10.1371/journal.pone.0023386 (PMC3158084; doi:10.1371/journal.pone.0023386)

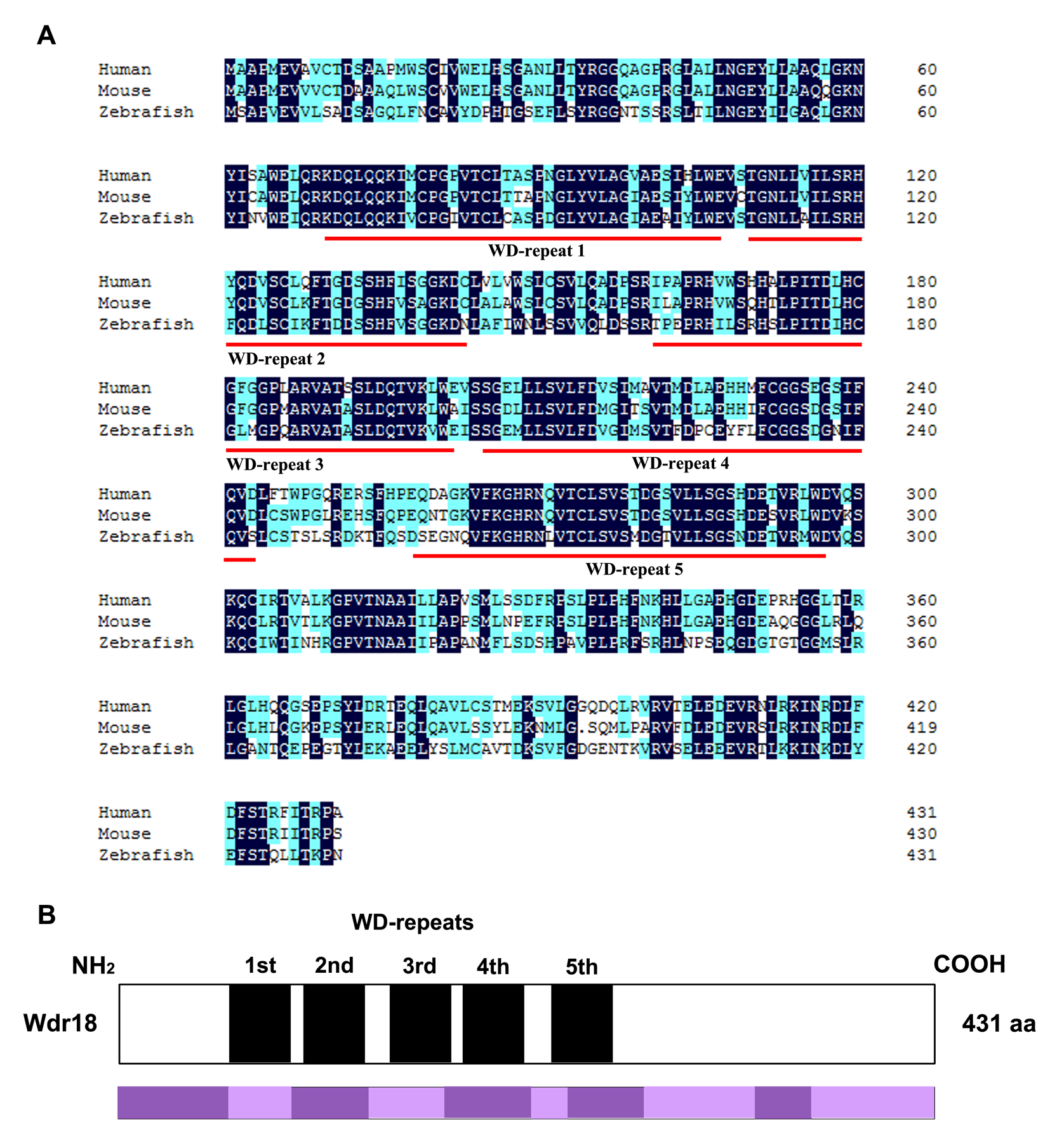

Supplement: Figure S1 — Sequence alignment and structure of Wdr18 protein. (A) Alignment of human, mouse and zebrafish Wdr18 peptide sequences. The amino acid residues that are identical in all three species are shaded in dark blue and those conserved in just two of them are shaded in light blue. Red horizontal lines below the sequences indicate the location of WD-repeats. (B) The schematic structure of zebrafish Wdr18 protein with black boxes indicating the location of the five WD repeats. The alternating dark purple and light purple boxes below indicate coding regions of exons 1–10 and the relative positions to the five WD-repeats. (TIF) [file pone.0023386.s001.tif]

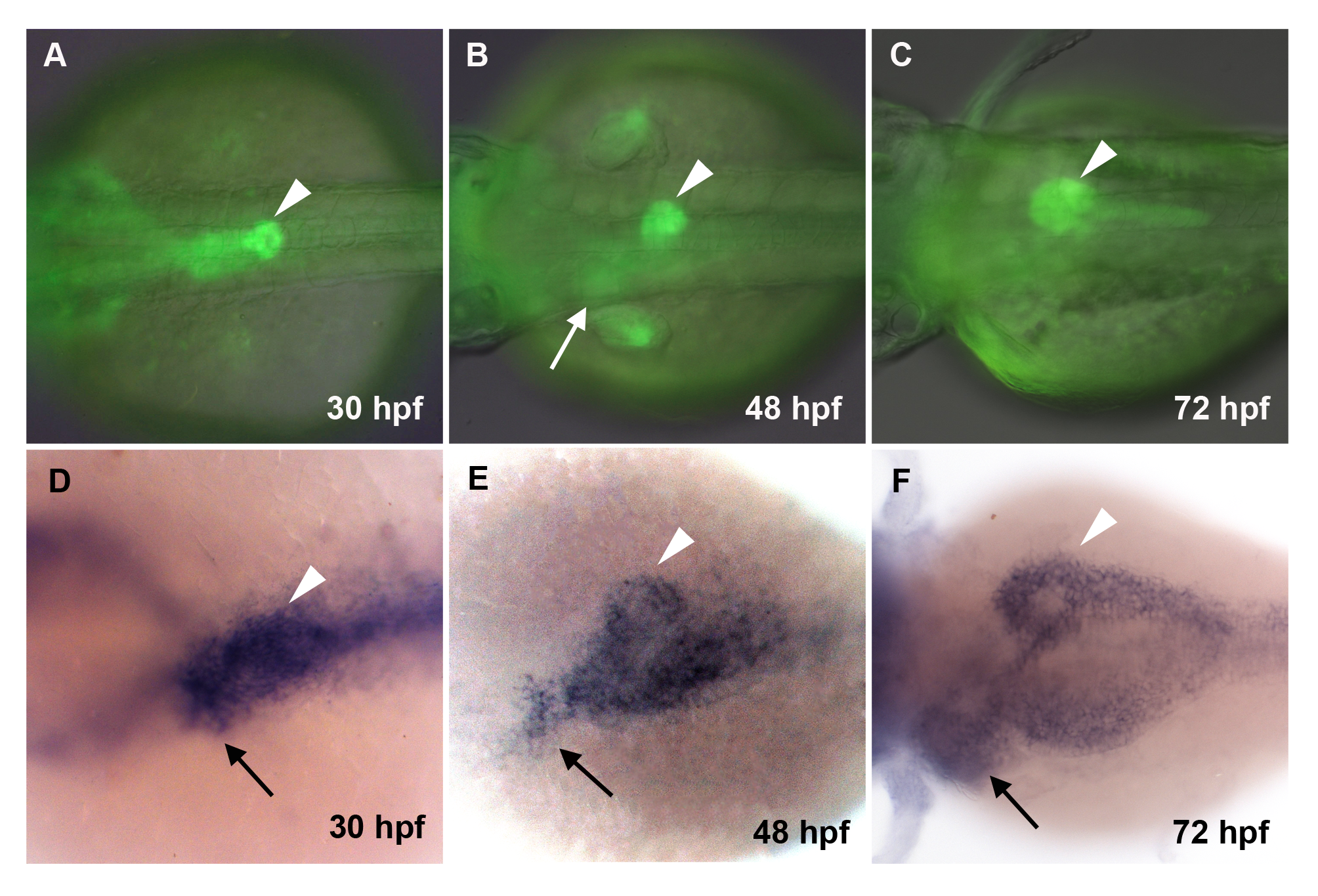

Supplement: Figure S2 — GFP fluorescent signal in mp235b embryos and wdr18 expression in endodermal organs. (A–C) GFP expression pattern in mp235b transgenic line. (D–F) Whole-mount in situ hybridization showing wdr18 gene expression in endodermal region during the first 3 days of development. Arrowheads indicate pancreas and arrows indicate liver region. All the embryos were shown as dorsal views with anterior to the left. (TIF) [file pone.0023386.s002.tif]

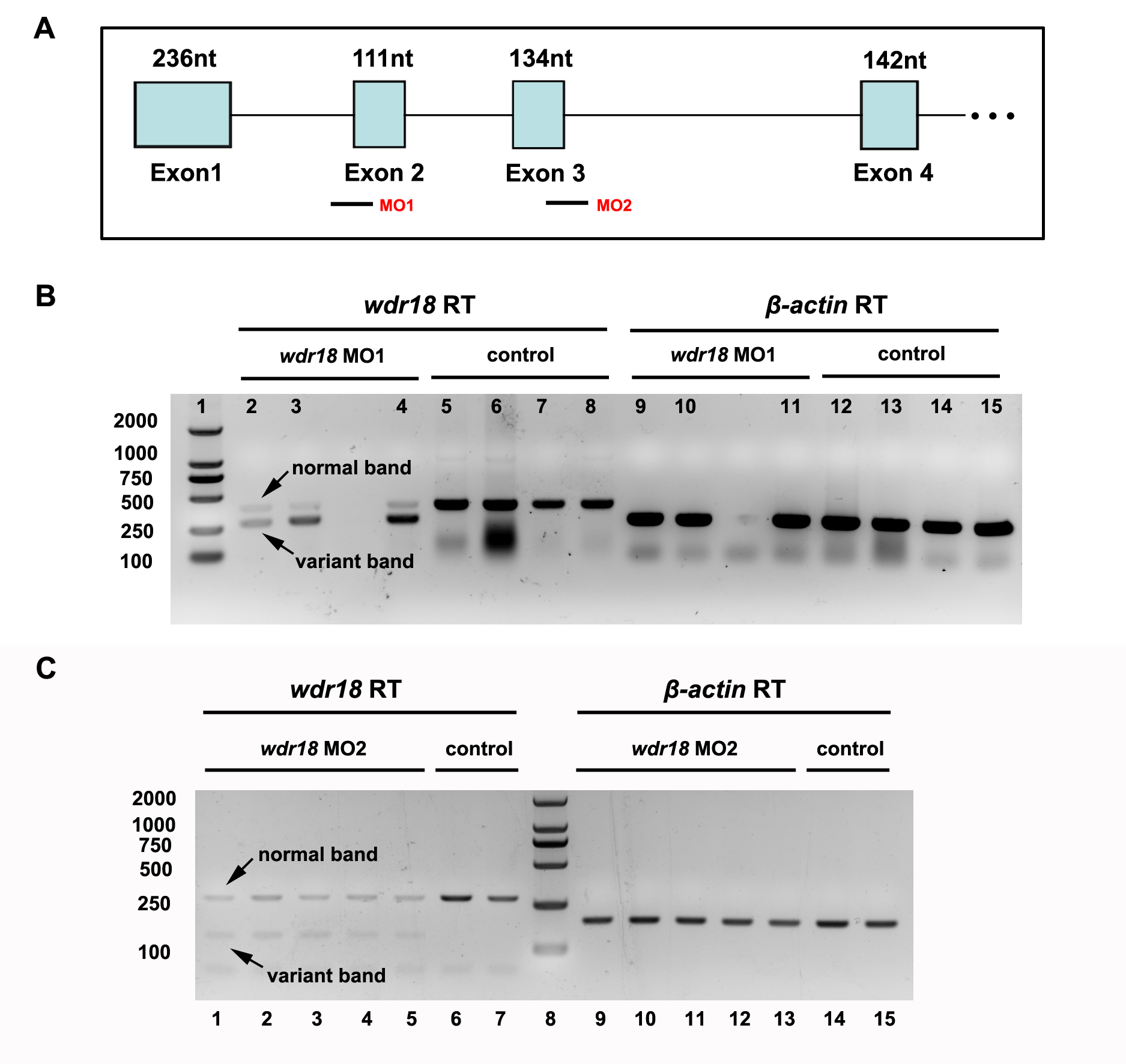

Supplement: Figure S3 — Injection of MO1 and MO2 against wdr18 result in efficient exon loss and mRNA down-regulation. (A) Illustration of the structure of wdr18 from 1st to 4th exons and the target sites for MO1 and MO2. (B) Introducing MO1 into embryos result in exon 2 skipping, or an 111 nt deletion of wdr18 mRNA, which causes the loss of the first WD repeat of Wdr18 protein. (C) Injection of MO2 leads to exon 3 skipping, or a 134 nt deletion, causing a frameshift for the sequence after exon 3. The total RNA were extracted from embryos at 1 dpf. (TIF) [file pone.0023386.s003.tif]

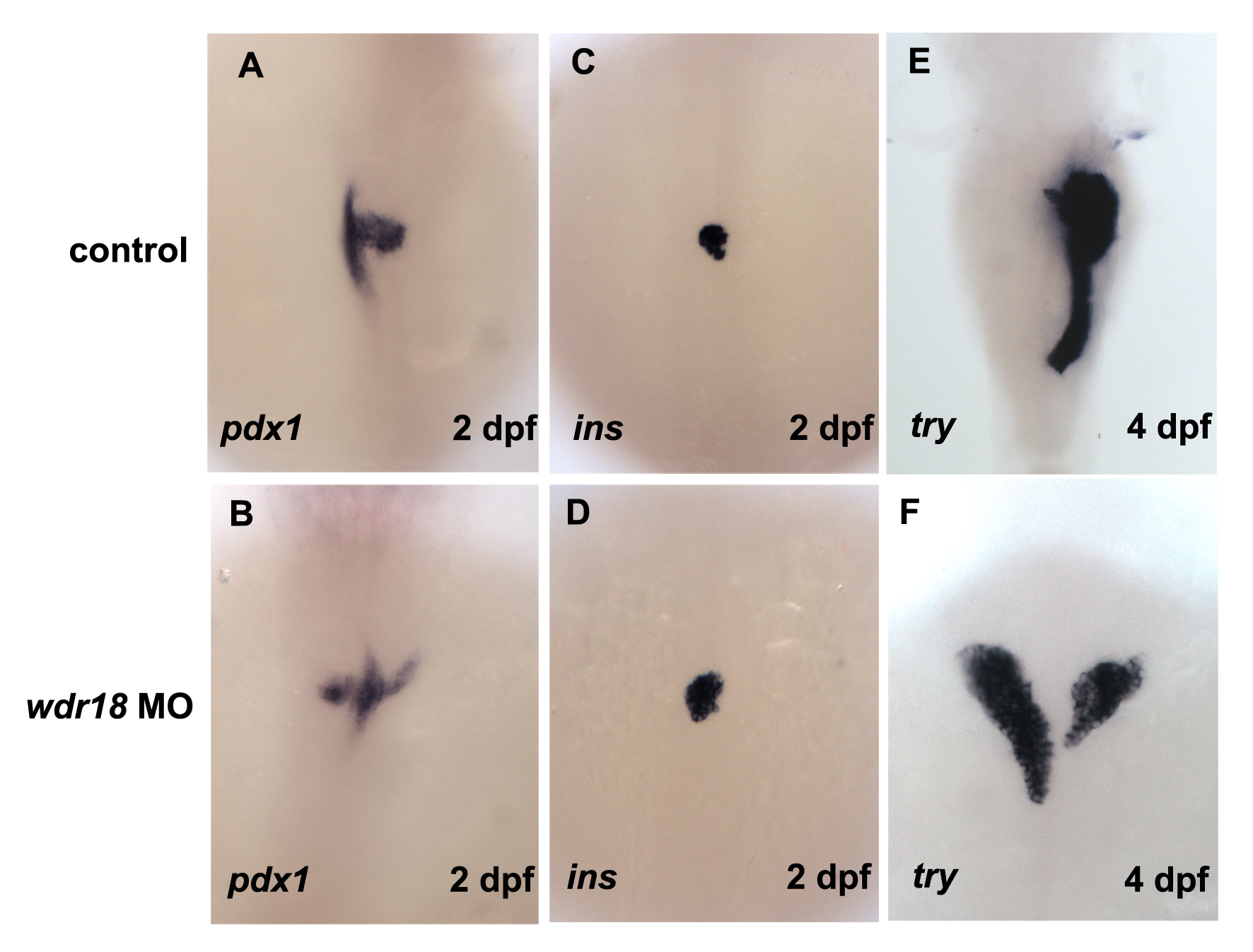

Supplement: Figure S4 — The effect of wdr18 knockdown on endodermal organs. (A, B) pdx1 was used as a marker for the pancreatic bud. In morphant embryos, the pancreas appeared on both sides of the body (B), while in control embryos it resided on the right side. (C, D) insulin (ins) was used as a differentiation marker for pancreatic β cells. Note that the development of β cells was not affected after wdr18 morpholino injection. (E, F) In situ hybridization of trypsin (try) revealing the exocrine pancreas at 4 dpf. In morphant embryos, the exocrine pancreas differentiated normally, but appeared in duplicated form. Dorsal views, anterior to the top. (TIF) [file pone.0023386.s004.tif]

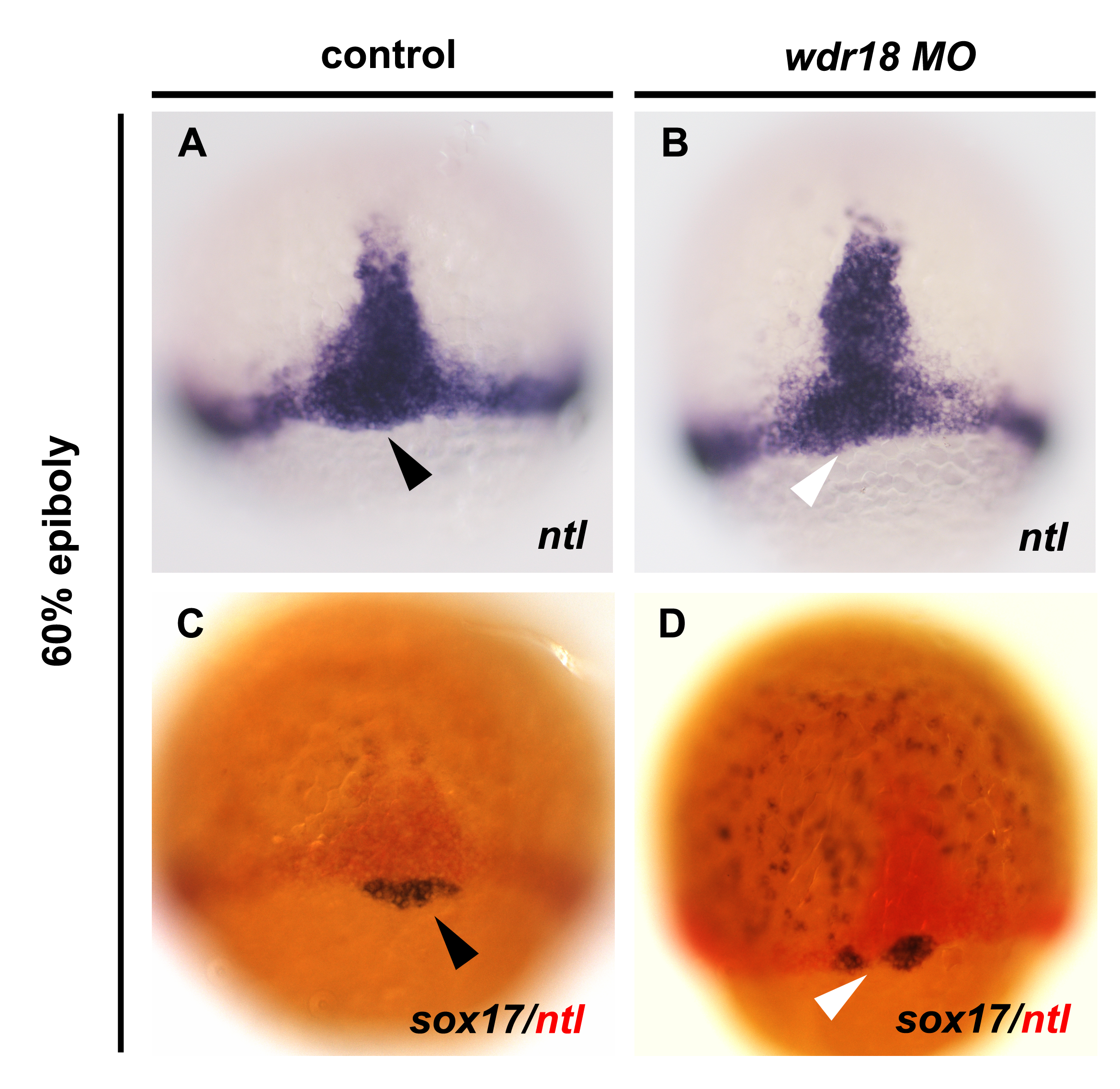

Supplement: Figure S5 — Knockdown of wdr18 led to defects in DFC migration without affecting DFC specification. (A) In situ hybridization result of ntl, showing the notochord precursor cells at the midline, marginal cells, and DFCs (black arrowhead) in a control embryo at 60%-epiboly stage. (B) The midline structure and marginal cells were not affected, while the DFC region, marked by a white arrowhead, seemed reduced or mis-localized in a wdr18 morphant. (C, D) Double in situ hybridization result showed clear staining of both ntl (red) and sox17 (blue) positive cells in wdr18 morphant embryos, indicating DFCs were still present after knockdown of wdr18, although they were not properly organized as a single cluster. Dorsal views, animal pole to the top. (TIF) [file pone.0023386.s005.tif]

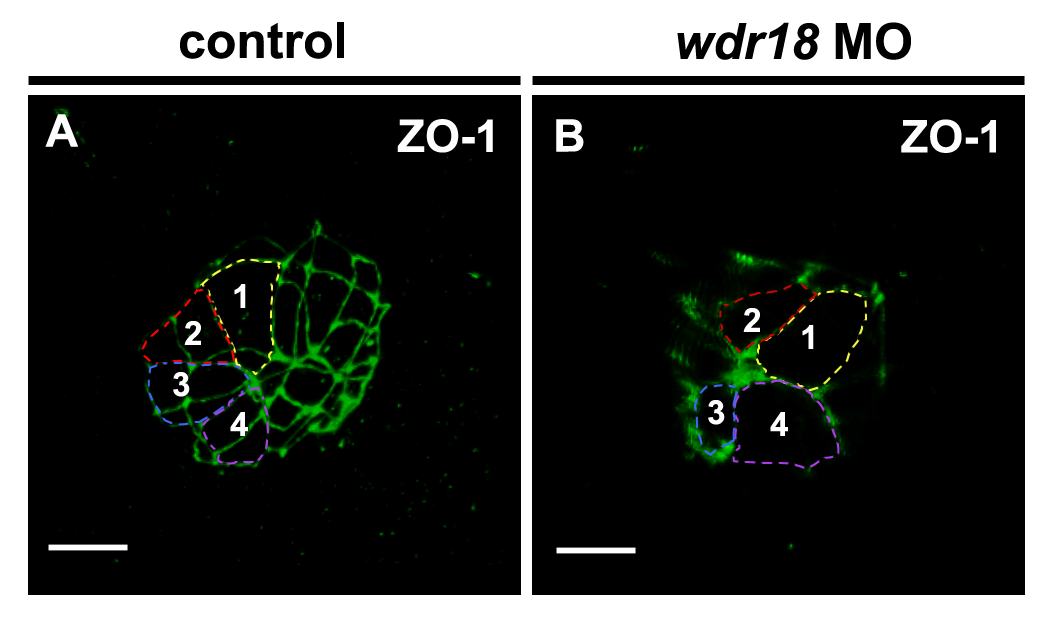

Supplement: Figure S6 — Knockdown of wdr18 did not change the size of cells composing Kupffer's vesicle. 3D reconstruction confocal images of the KV in control and wdr18 morphant embryos revealed by immuno-staining with ZO-1 antibody at 6-somite stage. For the convenience of outlining the cell shape, the 3D images were rotated to the proper position until the boundaries of the marked cells was clearly seen. (A) In a control embryo, the cell connection was obvious and we outlined four cells with different colors and numbered them from 1 to 4. (B) In a wdr18 morphant, we similarly outlined and numbered four cells for comparison. The size of the marked cells showed no obvious difference comparing with that in control embryos. Scale bars: 15 µm. (TIF) [file pone.0023386.s006.tif]
